# DNA barcoding and morphological analyses revealed validity of *Diadema clarki* Ikeda, 1939 (Echinodermata, Echinoidea, Diadematidae)

**DOI:** 10.3897/zookeys.585.8161

**Published:** 2016-04-27

**Authors:** Seinen Chow, Kooichi Konishi, Miyuki Mekuchi, Yasuji Tamaki, Kenji Nohara, Motohiro Takagi, Kentaro Niwa, Wataru Teramoto, Hisaya Manabe, Hiroaki Kurogi, Shigenori Suzuki, Daisuke Ando, Tadao Jinbo, Masato Kiyomoto, Mamiko Hirose, Michitaka Shimomura, Akira Kurashima, Tatsuya Ishikawa, Setuo Kiyomoto

**Affiliations:** 1 National Research Institute of Fisheries Science, 2-12-4 Fukuura, Yokohama, Kanagawa 236-8648, Japan; 2 Tokai University, Shizuoka, Shizuoka 424-8610, Japan; 3 Ehime University, Matsuyama, Ehime 790-8566, Japan; 4 Yokosuka Laboratory, National Research Institute of Fisheries Science, Kanagawa 238-0316, Japan; 5 Minami-Izu Laboratory, National Research Institute of Aquaculture, Shizuoka 415-0156, Japan; 6 Shibushi Laboratory, National Research Institute of Aquaculture, Kagoshima 899-7101, Japan; 7 Marine and Coastal Research Center, Ochanomizu University, Tateyama, Chiba 294-0301, Japan; 8 Kitakyushu Museum of Natural History and Human History, Kitakyushu, Fukuoka 805-0071, Japan; 9 Mie University, Tsu, Mie 514-8507, Japan; 10 Seikai National Fisheries Research Institute, Nagasaki, Nagasaki 851-2213, Japan

**Keywords:** Diadema clarki, Diadematidae, DNA barcoding, Echinoidea

## Abstract

A long-spined sea urchin *Diadema*-sp reported from Japanese waters was genetically distinct from all known *Diadema* species, but it remained undescribed. Extensive field surveys in Japan with molecular identification performed in the present study determined five phenotypes (I to V) in *Diadema*-sp according to the presence and/or shape of a white streak and blue iridophore lines in the naked space of the interambulacral area. All phenotypes were distinct from *Diadema setosum* (Leske, 1778) and *Diadema savignyi* (Audouin, 1829), of which a major type (I) corresponded to *Diadema clarki* Ikeda, 1939 that was questioned and synonymized with *Diadema setosum* by Mortensen (1940). The holotype of *Diadema clarki* has not been found, but three unlabeled dried tests of *Diadema* were found among Ikeda’s original collection held in the Kitakyushu Museum of Natural History and Human History, Fukuoka, Japan. A short mtDNA *COI* fragment (ca. 350bp) was amplified from one of the tests, and the nucleotide sequence determined (275bp) was nearly identical with that of *Diadema*-sp. Arrangements of the primary tubercles on the coronal plates in *Diadema*-sp and the museum specimen also conformed with *Diadema clarki*, indicating that *Diadema*-sp is identical to *Diadema clarki* and a valid species. Narrow latitudinal distribution (31°N to 35°N) of *Diadema clarki* in Japan was observed, where it co-existed with abundant *Diadema setosum* and rare *Diadema savignyi*. No *Diadema clarki* was found in the southern islands in Japan, such as Satsunan Islands to Ryukyu Islands and Ogasawara Island, where *Diadema setosum* and *Diadema savignyi* were commonly observed.

## Introduction

Long-spined sea urchins of the genus *Diadema* Gray, 1825 are abundant, widespread and ecologically important species in tropical to temperate areas (Muthiga and McClanahan 2007). Morphological similarity among *Diadema* species has made systematics a difficult task (Clark 1925, Mortensen 1940, Lessios et al. 2001, Muthiga and McClanahan 2007). Although Mortensen (1940) recognized six extant species in this genus, *Diadema antillarum* Philippi, 1845, *Diadema ascensionis* Mortensen, 1909, *Diadema mexicanum* A. Agassiz, 1863, *Diadema paucispinum* A. Agassiz, 1863, *Diadema savignyi* (Audouin, 1829), and *Diadema setosum* (Leske, 1778), considerable room for systematic revision has remained. Ikeda (1939) described a new species of *Diadema* from Japan under the name *Diadema clarki*, but Mortensen (1940) synonymized this new species with *Diadema setosum*. Baker (1967) added a new species *Diadema palmeri* Baker, 1967 from the north coast of New Zealand, and Pawson (1978) demoted *Diadema ascensionis* to a subspecies of *Diadema antillarum*. Advancements in molecular genetic analyses have shed further light on *Diadema* systematics, in which Lessios et al. (2001) using mitochondrial DNA (mtDNA) sequence analysis reported that *Diadema ascensionis* was nested within *Diadema antillarum*. Lessios et al. (2001) also detected substantially divergent sub-clades within *Diadema antillarum*, *Diadema paucispinum* and *Diadema setosum*, which strongly suggest the presence of cryptic species within the nominal species. Rodríguez et al. (2013) using mtDNA and morphological analyses raised eastern Atlantic population of *Diadema antillarum* to a new species *Diadema africanum* Rodríguez et al. 2013, which corresponds to the *Diadema antillarum*-b sub-clade reported by Lessios et al. (2001). Lessios et al. (2001) further found a genetically distinct species among specimens originally identified as *Diadema savignyi* or *Diadema setosum* in Japan and Marshal Islands, and tentatively designated them as *Diadema*-sp.

Recently, Chow et al. (2014) analyzed mtDNA of *Diadema savignyi*-like individuals from Sagami Bay (Kanagawa Prefecture, Pacific side) and Iki Island (Nagasaki Prefecture, Japan Sea side) in Japan and found these had the same mtDNA sequence as those that Lessios et al. (2001) called *Diadema*-sp. Considering the similar geographic origin, Lessios et al. (2001) suspected that *Diadema*-sp might be *Diadema clarki* Ikeda, 1939. Ikeda (1939) proposed the conspicuous white streaks running along the interambulacral zones and the arrangement of interambulacral tubercles to be diagnostic characteristics of *Diadema clarki*, which corresponded to those of *Diadema*-sp observed by Chow et al. (2014). Ikeda (1939) mentioned that “The type specimen is kept in the Zoological Laboratory, Kyushu Imperial University”, but he gave no further deposition information on the type specimen of *Diadema clarki*. All of Ikeda’s collections were not maintained at the laboratory, and we found meanwhile that the collection was moved to the Kitakyushu Museum of Natural History and Human History, Fukuoka, Japan. It was unfortunate that the labels of large number of specimens seemed to have been lost upon transfer, and three dried tests of *Diadema* found in the Ikeda’s original collection were not the exception. However, a short DNA fragment was amplified from one of these tests, and hence this dried test was utilized as a reference specimen.

In this study, molecular and phenotypic evidence are provided that *Diadema clarki* is *Diadema*-sp and hence a valid species, and we report the geographic distribution of *Diadema clarki* based on extensive field surveys.

## Materials and methods

**Figure 1.**
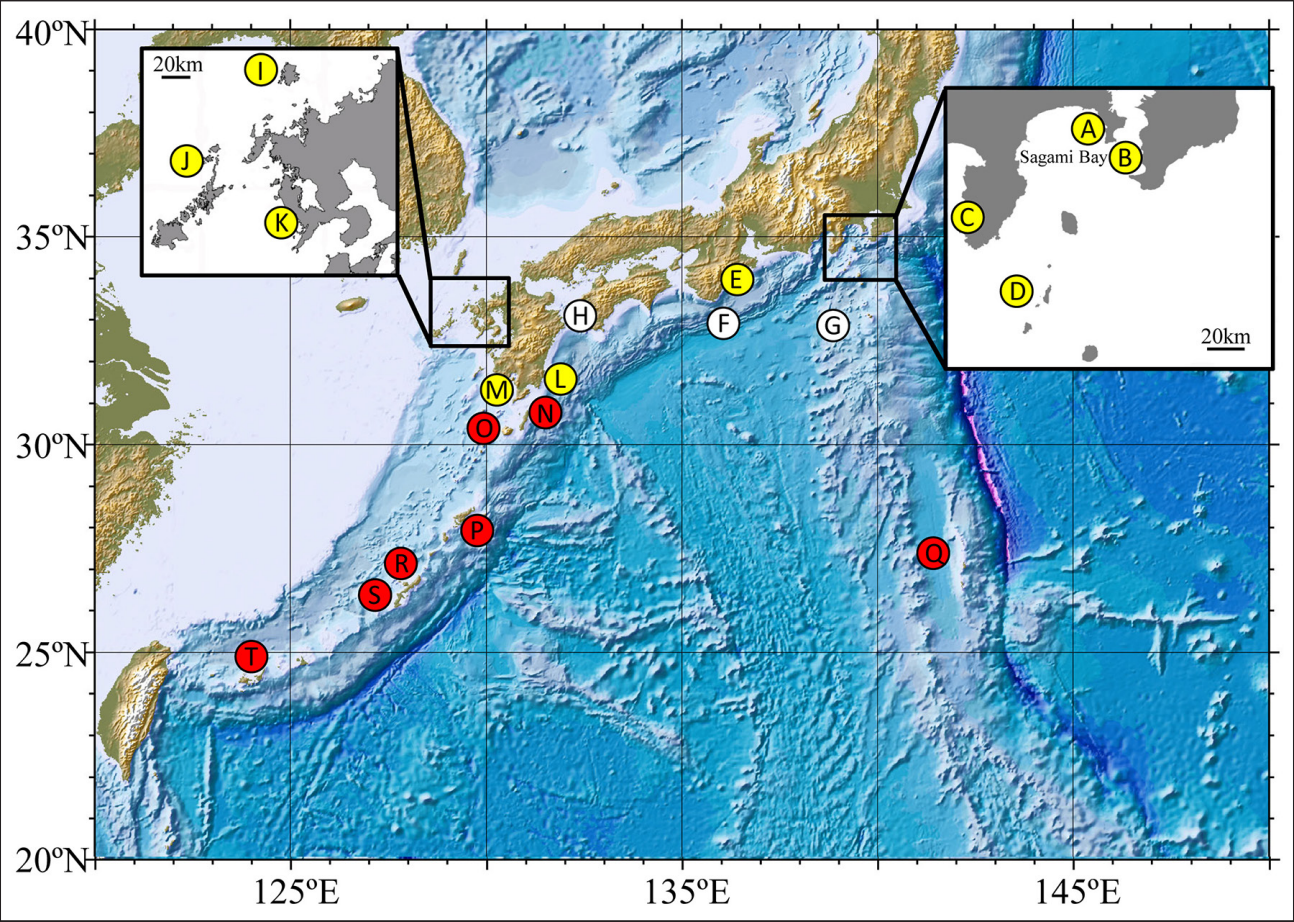
Localities where field observation and sampling of *Diadema* were performed. See Table 1 for detailed information. **A** Arasaki (Kanagawa Prefecture) **B** Tateyama (Chiba) **C** Mera (Shizuoka) **D** Shikine-jima (Tokyo) **E** Haida-ura (Mie) **F** Kushimoto (Wakayama) **G** Hachijo-jima (Tokyo) **H** Uchidomari (Ehime) **I** Iki-no-shima (Nagasaki) **J** Ojika (Nagasaki) **K** Mie (Nagasaki) **L** Shibushi (Kagoshima) **M** Kaimon (Kagoshima) **N** Tanega-shima (Kagoshima) **O** Yaku-shima (Kagoshima) **P** Amami Ohshima (Kagoshima) **Q** Ogasawara (Tokyo) **R** Motobu (Okinawa) **S** Sesoko (Okinawa) **T** Ishigaki-jima (Okinawa). *Diadema setosum* was observed in all areas surveyed. No *Diadema savignyi* but *Diadema*-sp were observed at localities with yellow circle, Both *Diadema savignyi* and *Diadema*-sp were observed at localities with white circle, No *Diadema*-sp but *Diadema savignyi* were observed at localities with red circle.

The twenty localities where field observations and/or collecting of *Diadema* specimens were carried out in Japanese waters are shown in Figure 1A–T. Based on the phenotypes to discriminate among *Diadema setosum*, *Diadema savignyi* and *Diadema*-sp as described in Chow et al. (2014), we selected *Diadema* individuals possessing characteristics neither of *Diadema setosum* nor *Diadema savignyi*. Although orange ring on the anal cone and white spots in naked space of the interambulacral areas are known to be characteristics of *Diadema setosum*, we found some individuals having the orange ring but no white spot during present survey. These “unusual” individuals were also determined to be *Diadema*-sp. Detailed locality information are presented in Table 1. Since many *Diadema*-sp might have been miss-identified as *Diadema savingnyi* in Japan mainland (see Chow et al. 2014), we recorded the number of *Diadema*-sp and *Diadema savignyi* encountered during the field survey. A quantitative survey of the phenotype variants of *Diadema*-sp was attempted in samples from Kanagawa (Figure 1A), Mie (Figure 1E), Nagasaki (Figure 1I–K), and Kagoshima (Figure 1M) Prefectures. A monthly scuba diving survey has been performed in order to investigate abundance and fecundity of *Diadema* spp. in Kanagawa Prefecture. The *Diadema*-sp individuals collected were transferred to aquaria, in which phenotype variation was studied. In Mie, Nagasaki, and Kagoshima Prefectures, *Diadema*-sp individuals encountered during scuba or skin diving surveys were photographed, and phenotype variation was examined based on photograph images. *Ad hoc* photographing *in situ* or in aquarium was performed in other areas, using which species identification was attempted. Of four *Diadema savignyi* individuals found and photographed at Motobu in Okinawa Island (Figure 1R), three (designated as OK2 to OK4) were transferred to the laboratory for subsequent analysis. Data of *Diadema savignyi* from Sesoko in Okinawa Island and Ishigaki-jima (Figure 1S and T) were obtained from previous study (Chow et al. 2014).

**Table 1.** Locality information for field survey and number of *Diadema*-sp and *Diadema savignyi* observed.

Locality	Prefecture	Figure 1	Lat (N)	Long (E)	Date	n^†^
Arasaki	Kanagawa	A	35°11'50"	139°35'59"	Dec. 2011^‡^ March-Sep. 2014	>400:0
Tateyama	Chiba	B	34°59'26"	139°49'28"	March to June, 2014	3:0
Mera	Shizuoka	C	34°39'39"	138°47'10"	May 2 and 23, 2015	22:0
Shikine-jima	Tokyo	D	34°19'13"	139°13'11"	Aug. 6, 2015	21:0
Haida-ura	Mie	E	33°59'48"	136°15'39"	March 10, 2012; April 15, 2015	70:0
Kushimoto	Wakayama	F	33°28'33"	135°44'29"	Sep. 29, 2014	1:3
Hachijo-jima	Tokyo	G	33°05'53", 33°07'20"	139°46'30", 139°49'00"	Feb. 26, 2007 July 30-31, 2015	0:1 4:22
Uchidomari	Ehime	H	32°56'31"	132°29'14"	Oct. 26, 2014	9:1
Iki-no-shima	Nagasaki	I	33°44'58"	129°38'56"	Sep. 2, 2014	37:0
Ojika	Nagasaki	J	33°11'05"	129°04'21"	July 19, 2014	102:0
Mie	Nagasaki	K	32°48'	129°45'	May 30, 2014	36:0
Shibushi	Kagoshima	L	31°27'55"	131°08'13"	May 17, 2014	2:0
Kaimon	Kagoshima	M	31°10'39"	130°33'14"	Oct. 22, 2014	19:0
Tanega-shima	Kagoshima	N	30°49'	131°02'	April 26, 2015	0:2
Yaku-shima	Kagoshima	O	30°27'	130°30'	Feb. 10, 2004	0:2
Amami Ohshima	Kagoshima	P	28°24'12"	129°27'15"	July 29, 2008	0:4
Ogasawara	Tokyo	Q	27°05'44"	142°11'58"	June 21, 2015	0:26
Motobu	Okinawa	R	26°39'16"	127°52'44"	July 16, 2014	0:4
Sesoko	Okinawa	S	26°38'09"	127°51'55"	May, 2013^‡^	0:4
Ishigaki-jima	Okinawa	T	24°27'	124°12'	Oct., 2013^‡^	0:7
Tulamben, Bari	(Indonesia)	not shown	8°16'29" ^§^	115°35'40"	March 8, 2015	0:2

† Number of individuals (*Diadema*-sp: *Diadema savignyi*) observed. ‡ Data from Chow et al. (2014). § Southern hemisphere.

Six individuals of *Diadema*-sp (designated as AT1 to AT3, AR54, AR59, AR70) showing phenotypic variation were chosen among the specimens collected at Arasaki during March to August 2014 (Table 2) and photographed in an aquarium. Tube feet of these six specimens collected in Arasaki along with those of three *Diadema savignyi* collected at Motobu (Table 2) were preserved in 70 % ethanol. Remaining bodies were fixed in neutralized 10% formaldehyde-sea water solution for two days, rinsed in running tap water overnight and transferred to 80 % ethanol. These samples were transferred to 70 % ethanol several months later and deposited to the Kanagawa Prefectural Museum of Natural History, Odawara, Kanagawa, Japan (Table 2). Three dried tests of *Diadema* were found among Ikeda’s original collection held in the Kitakyushu Museum of Natural History and Human History, but these tests were accompanied with no label and we numbered them as IK1 to IK3 (Table 2). Considering research field of Prof. Ikeda, these specimens were likely from Kumamoto, Nagasaki or Fukuoka Prefectures, all in Kyushu, Japan. Pieces of dried tissue remains from the base of spines or from the corona were collected and preserved in 70% ethanol.

**Table 2.** Information of five phenotypes (I to V) of six *Diadema*-sp individuals collected in Arasaki area, three museum specimens.

Sample	Voucher	Phenotype	Test size (mm)		Collection			GenBank
No.			diameter	height	locality	date	depth (m)	Accession No.
AT1	KPM-NJL000035	I	42.0	22.4	Arasaki	Mar. 24, 2014	2	LC037355
AT2	KPM-NJL000036	II	46.5	19.6	Arasaki	Mar. 24, 2014	3	LC037356
AT3	KPM-NJL000037	III	54.0	22.2	Arasaki	Mar. 24, 2014	2	LC037357
AR59	KPM-NJL000039	IV	44.0	19.2	Arasaki	July 28, 2014	2	LC037358
AR54	KPM-NJL000038	V	67.0	32.6	Arasaki	June 25, 2014	3	LC037359
AR70	KPM-NJL000040	V	53.8	20.8	Arasaki	Aug. 29, 2014	2	LC037360
IK1	KMNH IvR 500879	-	88.5	38.6	Kyushu	1933 or 1934	-	-
IK2	KMNH IvR 500880	-	65.4	36.9	Kyushu	1933 or 1934	-	-
IK3	KMNH IvR 500788	-	64.2	31.6	Kyushu	1933 or 1934	-	LC037361
OK2	KPM-NJL000041	-	91.7	38.6	Motobu	July 16, 2014	2	LC037362
OK3	KPM-NJL000042	-	64.0	31.5	Motobu	July 16, 2014	2	LC037363
OK4	KPM-NJL000043	-	40.0	19.5	Motobu	July 16, 2014	2	LC037364

All tissues fixed in ethanol and extracted DNA are kept in the National Research Institute of Fisheries Science, Kanagawa, Japan.

### Molecular analysis

Crude DNAs were extracted from the ethanol tissues preserved in ethanol and used for PCR amplification. In addition to the primers (COI120F and COI1300R) previously described (Chow et al. 2014), three internal primers (COI330F: 5’-TGATCAGTYTTTATCACCGC-3’; COI531F: 5’-ATGATTTCTCATGTAATTGC-3’; COI874R: 5’-AGTACAACGTCTATAGATGA-3’) were designed and used in this study. PCR amplification, nucleotide sequencing and phylogenetic analysis were performed as described in Chow et al. (2014).

## Results

### Phenotype analysis

#### *Diadema*-sp

**Figure 2.**
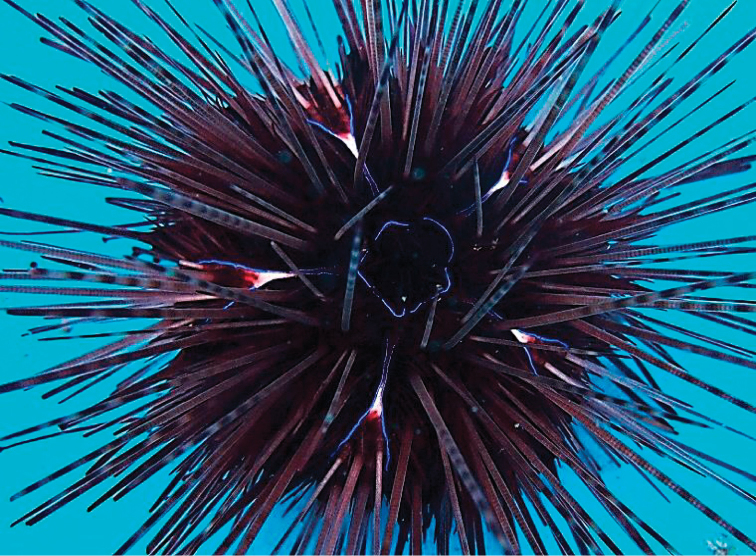
Underwater aboral view of phenotype I of *Diadema*-sp, KPM-NJL000035, original specimen number is AT1.

**Figure 3.**
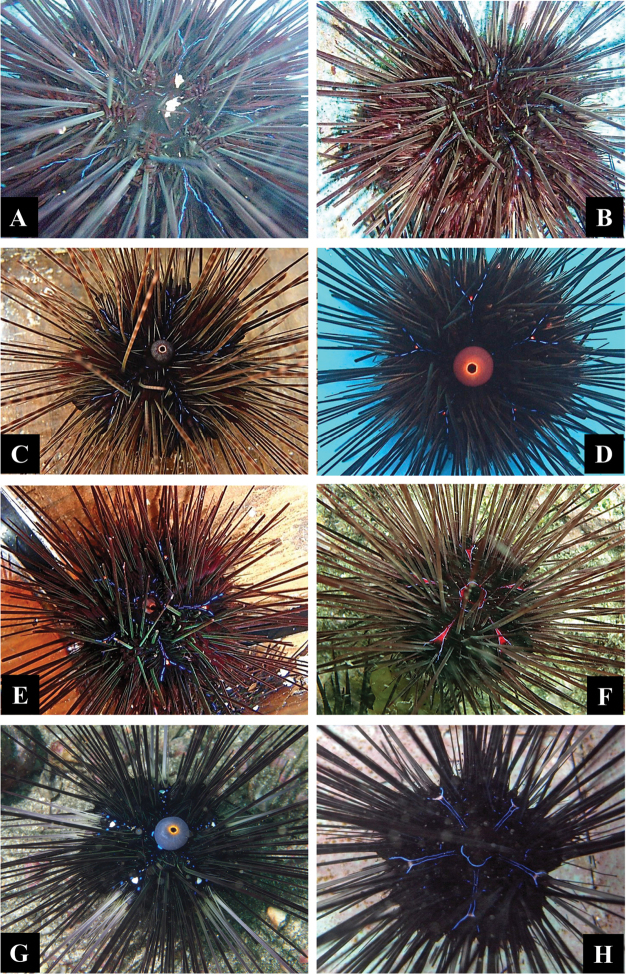
Underwater aboral views of four phenotypes (**A–E**) and color variants of phenotype I (**F**) of *Diadema*-sp, *Diadema setosum* (**G**), and *Diadema savignyi* (**H**). **A** KPM-NJL000036, original specimen number is AT2 designated as phenotype II **B** KPM-NJL000037, original specimen number is AT3 designated as phenotype III **C** KPM-NJL000039, original specimen number is AR59 designated as phenotype IV **D** KPM-NJL000038, original specimen No. is AR54 designated as phenotype V **E** KPM-NJL000040, original specimen No. is AR70 designated as phenotype V **F** color variant of phenotype I observed at Haida-ura in Mie Prefecture (Figure 1E) photographed by T. Ishikawa **G** *Diadema setosum* (DST2 in Chow et al. (2014)) **H** *Diadema savignyi* (DSV23 in Chow et al. (2014)).

Underwater images of several phenotypes in *Diadema setosum*, *Diadema savignyi* and *Diadema*-sp were presented in Chow et al. (2014). Phenotype variants in *Diadema*-sp observed in the present study and reference images of *Diadema setosum* and *Diadema savignyi* obtained in the previous study (Chow et al. 2014) are shown in Figures 2 and 3. *Diadema setosum* had characteristics of five white spots in naked space of the interambulacral areas and orange ring on the anal cone (Figure 3G). Many individuals of this species had small blue iridophore dots aligned along the naked space of the interambulacral areas. *Diadema savignyi* had characteristics of Y-shaped blue iridophore lines (YBIL) running along the naked space of the interambulacral areas and no orange ring on the anal cone (Figure 3H). Some *Diadema savignyi* had small white crescent at the fork region of YBIL but never resembled white spot of *Diadema setosum*. Three phenotypes in *Diadema*-sp were reported in Chow et al. (2014), corresponding to those presented in Figure 2 and Figure 3A, B. YBIL shape of *Diadema*-sp was substantially different from that of *Diadema savignyi* (see also Chow et al. 2014). We have never observed *Diadema savignyi* in monthly survey performed at Arasaki area since 2011, but encountered a few *Diadema* individuals having orange ring on the anal cone with orange spot at the fork region of YBIL but no white spot. We here determined five phenotype variants (I to V) (Figure 2 and Figure 3A–F) in *Diadema*-sp. Phenotype I was the most common, having conspicuous white streak at the fork region of the intact YBIL (Figure 2), corresponding to the description of *Diadema clarki* by Ikeda (1939). The other phenotypes had no white streak. YBIL of phenotype II was intact (Figure 3A), while that in the other phenotypes was broken (Figure 3B–E). Phenotype III had broken YBIL (Figure 3B). Phenotype IV had orange ring on the anal cone (Figure 3C). Phenotype V was similar to phenotype IV but had small orange dot at the fork region of broken YBIL (Figure 3D, E). White streaks in some individuals were small (not shown) and some individuals had red streaks (Figure 3F), but all these variants were classified as phenotype I.

**Figure 4.**
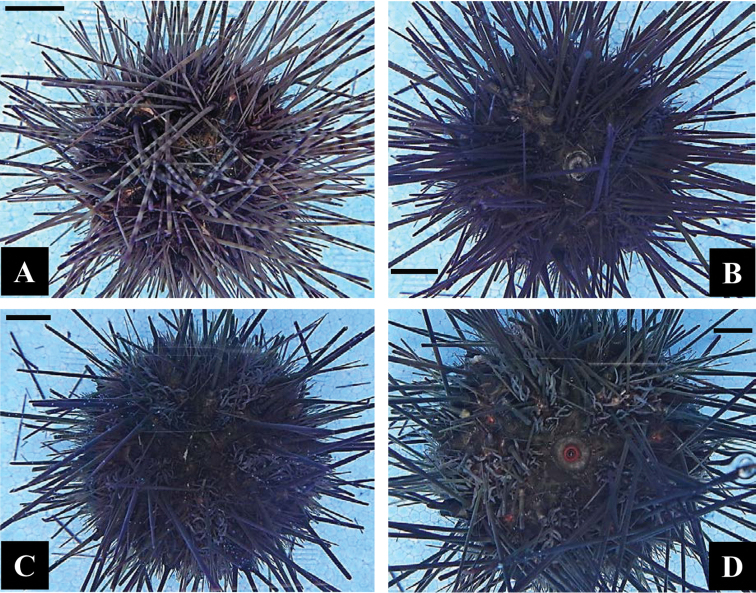
Preserved specimen of phenotypes I (**A**), II (**B**), III (**C**) and V (**D**) of *Diadema*-sp, corresponding to AT1 (KPM-NJL000035) in Figure 2, and AT2, AT3 and AR54 (KPM-NJL000036‒KPM-NJL000038) in Figures 3A, B and D, respectively. All scale bars = 10 mm.

These characteristics in living specimen conspicuous underwater were not well preserved after fixation (Figure 4). In the preserved specimens, YBILs of all phenotypes were completely disappeared, while white streak in phenotype I (Figure 4A) and orange ring and orange dot in phenotype V (Figure 4D) were barely seen.

### Museum specimens

**Figure 5.**
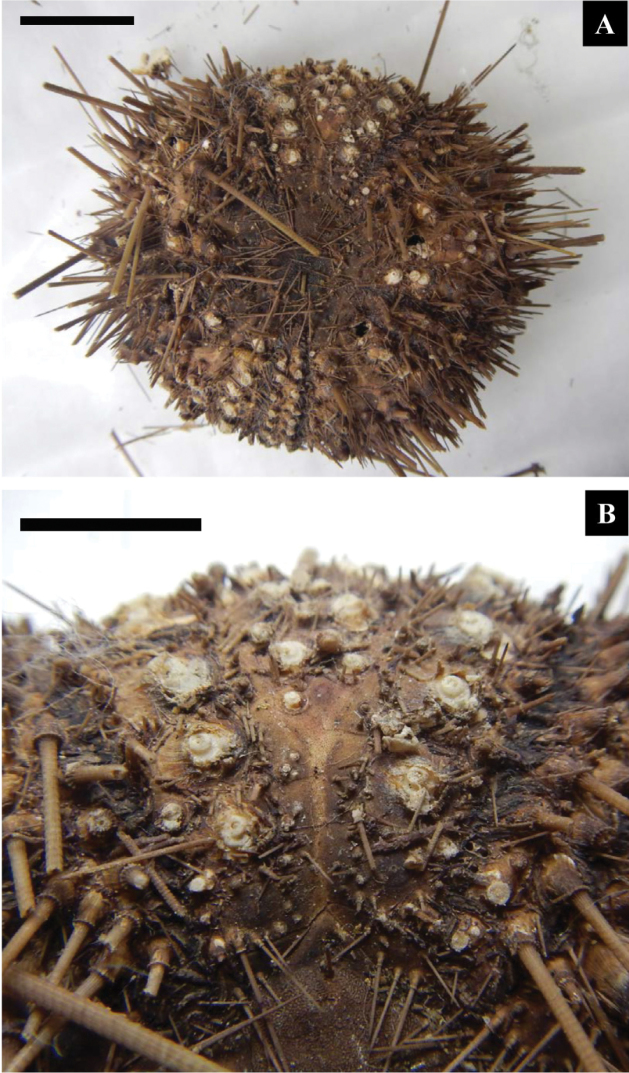
Aboral view (**A**) and enlarged view of a naked space of the interambulacral area (**B**) in a reference dried specimen of *Diadema* found in Ikeda’s collection. KMNH IvR 500788, original specimen No. is IK3. White streak-like remnant can be seen (**A, B**), and the outer and inner series of primary tubercles initiated on the 3^rd^ and 5^th^ coronal plates, respectively (**B**). Scales bars = 20 mm (**A**) and 10 mm (**B**).

Aboral views of the dried test of IK3 are presented in Figure 5. No PCR amplification was observed in the other museum specimens (IK1 and 2). A streak-like white line was observed on the naked space of the interambulacral areas, and the outer and inner series of primary tubercles initiated on the 3^rd^ and 5^th^ coronal plates, respectively (Figure 5B). These correspond to Ikeda’s (1939) description on *Diadema clarki* and to observations by Chow et al. (2014) on *Diadema*-sp.

### Genetic analysis of museum and field specimens of *Diadema*

**Figure 6.**
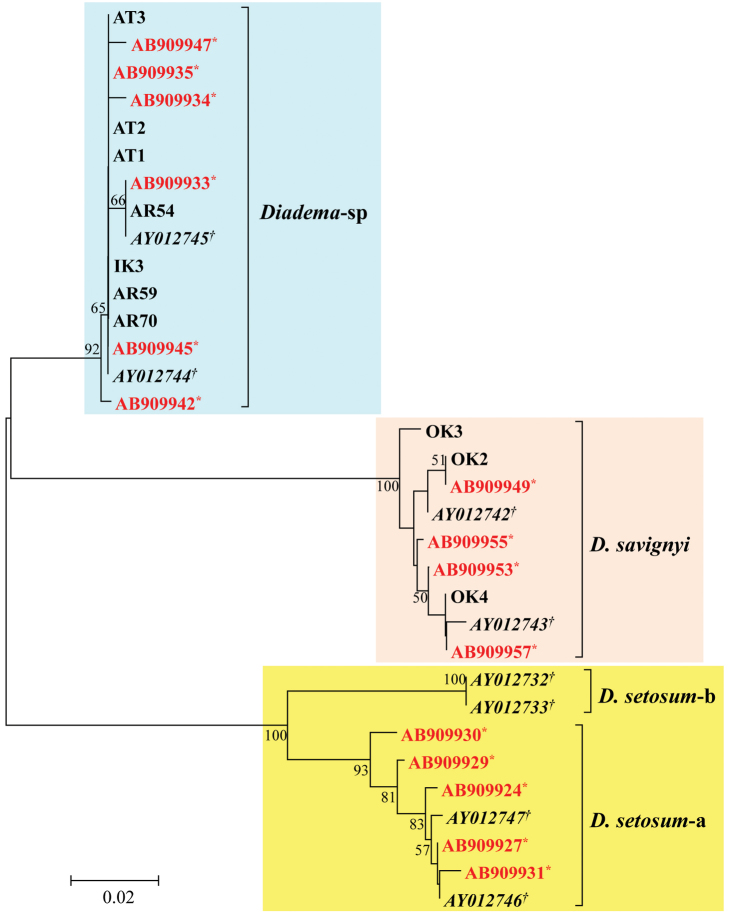
Neighbor-joining phylogenetic tree drawn using from *COI* sequence data. Bootstrap support (>50%) after 1,000 replications is shown at each node. Italic accession numbers with dagger (AY012732, AY012733, AY012742–AY012747) are from Lessios et al. (2001) and red accession numbers with asterisk (AB900024, AB909927, AB909929–AB909931, AB909933–AB909935, AB909942, AB909945, AB909947, AB909949, AB909953, AB909955, AB909957) are from Chow et al. (2014).

Approximately 1,100 bp fragments could be amplified in three *Diadema savignyi* (OK) and six *Diadema*-sp (AT and AR) individuals using a primer pair (COI120FxCOI1300R). All possible primer combinations were tested in three museum specimens (IK1 to IK3), but successful amplification (c.a. 350 bp fragment) was obtained only in IK3 by one primer pair (COI531FxCOI874R). Nested PCR was also attempted for the other museum specimens, but no amplification was observed. IK3 was therefore designated as a reference specimen of the Ikeda’s collection and deposited to the Kitakyushu Museum of Natural History and Human History (voucher: KMNH IvR 500788). Nucleotide sequences determined were 275 bp for IK3, 411 bp for AR samples, 440 bp for OK samples, and 944–950 bp for AT samples. These sequences are available in DDBJ/EMBL/GenBank database (LC037355 to LC037364). Using MEGA v6 (Tamura et al. 2013), these sequences were aligned with several sequences of *Diadema setosum*, *Diadema savignyi* and *Diadema*-sp previously published by Lessios et al. (2001) and Chow et al. (2014), in which the gamma-corrected Kimura’s two parameter (K2P) distance was selected as the best-fit model for nucleotide substitution. The phylogenetic analysis clearly indicates that specimen IK3 and the six *Diadema*-sp individuals collected in this study (AT and AR specimens) are clustered together within a unique clade, distinct from other clades (Figure 6). Mean nucleotide sequence divergences (K2P: pairwise deletion option) within and between species are presented in Table 3. Average K2P between IK3 and *Diadema*-sp was 0.16±0.09%, which was well within the intraspecific divergence values of *Diadema* (see Lessios et al. 2001, Chow et al. 2014). This estimate is much smaller than those between IK3 and *Diadema savignyi* (12.06±2.35%), IK3 and *Diadema setosum*-a (13.13±2.84%), and IK3 and *Diadema setosum*-b (12.72±2.80%). These indicate that all *Diadema*-sp phenotypes and IK3 are conspecific.

**Table 3.** Mean percent nucleotide sequence divergence (K2P±SE) within (on the diagonal) and between (below diagonal) species. Number of individuals within brackets.

	IK3	*Diadema*-sp	*Diadema savignyi*	*Diadema setosum*-a	*Diadema setosum*-b
IK3 (1)	-				
*Diadema*-sp (14)	0.16±0.09	0.26±0.09			
*Diadema savignyi* (9)	12.06±2.39	12.14±1.56	1.11±0.25		
*Diadema setosum*-a (7)	13.13±2.81	16.96±1.93	18.50±2.21	0.84±0.19	
*Diadema setosum*-b (2)	12.72±2.79	13.89±2.07	19.30±2.61	7.22±1.24	0.00±0.00

See Figure 6 for nucleotide sequences obtained from database.

### Ecology

*Diadema*-sp was seen in the subtidal zone, ranging to depths of 8 m but no further attempt was performed to investigate their distribution in deeper zones. Both *Diadema setosum* and *Diadema*-sp were observed in the same habitats and depths, but the former had tendency to aggregate and the later was usually seen as solitary specimens; in consequence both usually did not occur side by side. Although relative abundances of *Diadema setosum* and *Diadema*-sp were not quantitatively investigated, the former species was relatively abundant and ubiquitously observed throughout all the areas examined. However, after the severe winters of 2014 and 2015 (January to February), *Diadema*-sp were observed to be predominated at Arasaki area, suggesting that it may be more tolerant to cold water than *Diadema setosum*. In addition, *Diadema savignyi* may be less tolerant to lower temperatures than *Diadema setosum*, since *Diadema savignyi* was never found at Arasaki area and is not common around Japan mainland.

### Distribution and phenotype frequency

In contrast to the ubiquitous distribution of *Diadema setosum* throughout the survey areas, *Diadema*-sp was only observed in a narrow latitudinal range around Japan mainland, from Kanagawa (35°11’ N) to Kagoshima (31°10’ N) (see Figure 1 and Table 1). No *Diadema*-sp was found in the Satsunan Islands (Tanega-shima, Yaku-shima, and Amami Oshima) to the Ryukyu Archipelago (Figure 1N–P, R–T), nor in Ogasawara Island (Figure 1Q), where *Diadema setosum* and *Diadema savignyi* were commonly observed. *Diadema savignyi* was rare around Japan mainland and observed with *Diadema*-sp and *Diadema setosum* at Kushimoto in Wakayama (Figure 1F) and Uchidomari in Ehime (Figure 1H) (see also Table 1). The three species also co-existed in Hachijo-jima (Figure 1G), where *Diadema*-sp became a minority (Table 1).

Frequency of the five phenotypes observed in Kanagawa, Mie, Nagasaki and Kagoshima Prefectures is presented in Table 4. Phenotype I was most commonly observed at all localities and during all sampling days, followed by phenotypes II and III. Phenotypes IV and V were much less frequently observed. Frequency distribution of these phenotypes was found significantly different among sampling days at Arasaki area (χ^2^ test, P = 0.024) but not among three localities in Nagasaki (P = 0.089). Significant heterogeneity in the phenotype frequency was observed among pooled samples of the four Prefectures (P < 0.001).

**Table 4.** Relative abundance (percentage) of the five phenotypes of *Diadema*-sp observed in Kanagawa, Mie, Nagasaki and Kagoshima Prefectures.

Phenotype	Kanagawa			Mie	Nagasaki			Kagoshima
	Arasaki			Haida-ura	Iki Isl.	Ojika	Mie	Kaimon
	June 2014	July 2014	Sep. 2014	April 2015	Sep. 2014	July 2014	May 2014	Oct. 2014
I	64.1	58.8	71.4	73.1	64.6	52.6	38.9	52.6
II	14.8	4.1	7.7	20.9	17.7	21.1	30.5	21.1
III	12.0	27.8	15.4	3.0	17.7	10.5	16.7	15.8
IV	6.3	6.2	3.3	3.0	0.0	5.3	11.1	10.5
V	2.8	3.1	2.2	0.0	0.0	10.5	2.8	0.0
total (n)	142	97	91	67	34	57	36	19

## Discussion

The present investigation together with previous studies (Lessios et al. 2001, Chow et al. 2014) revealed phenotypic and genetic characteristics of *Diadema*-sp distinct from congeneric species (*Diadema setosum* and *Diadema savignyi*) occurring in Japan. The conspicuous white streak of phenotype I and arrangements of the outer and inner series of primary tubercles observed in *Diadema*-sp correspond to the description on *Diadema clarki* by Ikeda (1939). One of Ikeda’s specimen (IK3) was genetically identified to be *Diadema*-sp and the arrangements of the outer and inner series of primary tubercles were similar to *Diadema clarki*. Furthermore, the present distributions of *Diadema*-sp correspond to that of *Diadema clarki*. These indicate that *Diadema*-sp appears to be *Diadema clarki* and a valid species. Ikeda (1939) did not provide specific size data and museum repository numbers for his type specimens of *Diadema clarki*, but he only stated that the largest specimen was 65 mm in diameter among 22 individuals collected in 1933 and 1934. IK1 (88.5 mm in diameter) is too large to be in his type series, while IK2 and 3 (65.4 and 64.2 mm, respectively) could be. Aboral view photograph of a dried *Diadema clarki* test presented in Ikeda (1939) could not be compared with IK2 and 3, since it was of “smaller individual” (Ikeda 1939).

Among the several characters of *Diadema clarki* described by Ikeda (1939), the white or red streaks running along the interambulacrals may be the most distinguishing character from other species in living specimen. Ikeda (1939), however, did not mention any variation in the white streak appearance, and he might have considered only the phenotype I to be *Diadema clarki*. Size and shape of the white (or red) streak appear to vary (see also Chow et al. 2014), of which a smaller one might be miss-identified as white spot of *Diadema setosum* and individuals having no white streak might be miss-identified as *Diadema savignyi*. In fact, photographs shown as *Diadema savignyi* in previous reports (Shigei 1986, Kohtsuka 2005) are obviously of *Diadema clarki*. Although the tridentate pedicellariae may be a diagnostic characteristic for species identification in the genus *Diadema* (Coppard and Campbel 2006a), Mortensen (1940) and Ikeda (1939) both did not consider the tridentate pedicellariae of *Diadema clarki* to be specific characteristic for discriminating it from *Diadema setosum*. As Mortensen (1940) examined preserved specimens of *Diadema clarki*, the white streak might have been obscured and hence regarded to be a variant of diagnostic white spot of *Diadema setosum*. Although YBIL of *Diadema clarki* and *Diadema savignyi* and small blue iridophore dots of *Diadema setosum* may be better diagnostic keys for discriminating all these three species as already demonstrated by Coppard and Campbell (2006b), Ikeda (1939) did not mention this character at all. Characters not suitable for preservation might be neglected or unnoticed. Thus, underwater coloration images of living specimens are necessary for properly identifying *Diadema clarki*, although the white streak may occasionally remain observable even after preservation. These characteristics of *Diadema clarki*, distinct from *Diadema setosum* and *Diadema savignyi*, were noted for some of the samples collected from the Seto Marine Biological Laboratory, Shirahama, Japan, and used in Lessios et al. (2001), but specimens were assumed to be hybrids of *Diadema setosum* and *Diadema savignyi* (J.S. Pearse, pers. comm.).

Ikeda (1939) stated that all 22 *Diadema clarki* individuals observed had an orange ring at the end of the anal cone as in *Diadema setosum*, and it was observed that *Diadema clarki* individuals (phenotypes IV and V) have this same orange ring (Figure 3C–E) but much less frequently. There is another discrepancy between Ikeda (1939) and our observations: Ikeda (1939) described his *Diadema clarki* individuals to have the white streak and the orange ring together, but such a combination has never been observed by the authors. Assuming the phenotypic characters to be heritable, genetic drift may explain the change of a phenotype frequency. However, fixation of a phenotype combination in Ikeda’s time and the separation of these phenotypes at present time are unexplainable by genetic drift alone. The size or color variation in the white streak, orange ring and orange dot described in the present study might be partially an environment-associated character, which may be responsible for type frequency difference between localities or among sampling dates (Table 4).

Since experimental hybridization between *Diadema setosum* and *Diadema savignyi* produced viable hybrids (Uehara et al. 1990) and occurrence of natural hybrids between *Diadema setosum*, *Diadema savignyi* and *Diadema paucispinum* was reported by Lessios and Pearse (1996), hybridization between *Diadema clarki* and other species may not be ruled out. All phenotype variants of *Diadema clarki* had nearly identical *COI* sequences one another, but asymmetrical fertilization success may be the case for *Diadema clarki* as observed in strongylocentroid sea urchins (Addison and Pogson 2009). Since *Diadema clarki* was not recognized as a valid species and the phenotype variants were similar to some of the suspected hybrids reported by Lessios and Pearse (1996), it is highly probable that the suspected hybrids specifically from Shirahama, Japan, examined by them may be *Diadema clarki*. Nevertheless, genetic analysis on nuclear genome may be necessary for investigating occurrence of natural hybrids.

So far as published data, *Diadema clarki* is not observed in the Ryukyu Archipelago (Lessios et al. 2001, Chow et al. 2014), and no *Diadema clarki* were observed in Indonesia. On the other hand, the distribution was genetically confirmed in remote tropical islands such as Marshal Island (Lessios et al. 2001). Given that some of the suspected hybrids observed by Lessios and Pearse (1996) were *Diadema clarki*, the distribution could be much wider extending to Papua New Guinea and Indonesia.
